# Napsin A Expression in Human Tumors and Normal Tissues

**DOI:** 10.3389/pore.2021.613099

**Published:** 2021-04-20

**Authors:** Sören Weidemann, Jan Lukas Böhle, Hendrina Contreras, Andreas M. Luebke, Martina Kluth, Franziska Büscheck, Claudia Hube-Magg, Doris Höflmayer, Katharina Möller, Christoph Fraune, Christian Bernreuther, Michael Rink, Ronald Simon, Anne Menz, Andrea Hinsch, Patrick Lebok, Till Clauditz, Guido Sauter, Ria Uhlig, Waldemar Wilczak, Stefan Steurer, Eike Burandt, Rainer Krech, David Dum, Till Krech, Andreas Marx, Sarah Minner

**Affiliations:** ^1^ Institute of Pathology, University Medical Center Hamburg-Eppendorf, Hamburg, Germany; ^2^ Department of Urology, University Medical Center Hamburg-Eppendorf, Hamburg, Germany; ^3^ Institute of Pathology, Clinical Center Osnabrueck, Osnabrueck, Germany; ^4^ Department of Pathology, Academic Hospital Fuerth, Fuerth, Germany

**Keywords:** napsin A, immunohistochemistry, tissue micro array, diagnostic, human cancer types

## Abstract

**Background:** Novel aspartic proteinase of the pepsin family A (Napsin A, TAO1/TAO2) is a functional aspartic proteinase which is involved in the maturation of prosurfactant protein B in type II pneumocytes and the lysosomal protein catabolism in renal cells. Napsin A is highly expressed in adenocarcinomas of the lung and is thus commonly used to affirm this diagnosis. However, studies have shown that other tumors can also express Napsin A.

**Methods:** To comprehensively determine Napsin A expression in normal and tumor tissue, 11,957 samples from 115 different tumor types and subtypes as well as 500 samples of 76 different normal tissue types were evaluable by immunohistochemistry on tissue microarrays.

**Results:** Napsin A expression was present in 16 different tumor types. Adenocarcinoma of the lung (85.6%), clear cell adenocarcinoma of the ovary (71.7%), clear cell adenocarcinoma of the endometrium (42.8%), papillary renal cell carcinoma (40.2%), clear cell (tubulo) papillary renal cell carcinoma (16.7%), endometrial serous carcinoma (9.3%), papillary thyroid carcinoma (9.3%) and clear cell renal cell carcinoma (8.2%) were among the tumors with the highest prevalence of Napsin A positivity. In papillary and clear cell renal cell carcinoma, reduced Napsin A expression was linked to adverse clinic-pathological features (*p* ≤ 0.03).

**Conclusion:** This methodical approach enabled us to identify a ranking order of tumors according to their relative prevalence of Napsin A expression. The data also show that loss of Napsin A is linked to tumor dedifferentiation in renal cell carcinomas.

## Introduction

Novel aspartic proteinase of the pepsin family A (Napsin A, TAO1/TAO2) belongs to the peptidase A1 family, such as Cathepsin E, renin, and pepsin and is encoded by the *NAPSA* gene located at chromosome 19q13.3 [1–3]. Napsin A is a functional aspartic proteinase, harboring two aspartic acids inside the catalytical center that cleaves proteins and peptides to produce mature or active forms of these molecules [2, 3]. Napsin A is mainly expressed in the cytoplasm of type II pneumocytes, intra-alveolar macrophages, proximal and convoluted renal tubules, and pancreatic acini and ducts [4, 5] as well as adenocarcinomas of the lung, papillary renal cell carcinomas, and ovarian clear cell carcinomas [1, 6, 7]. Physiologically, Napsin A is involved in the maturation of prosurfactant protein B in type II pneumocytes [8], potentially in phagocytosis by macrophages [3] and the lysosomal protein catabolism in renal cells [9]. In addition, it was demonstrated that Napsin A is regulated by thyroid transcription factor 1 (TTF1), a diagnostic marker in lung cancer [3]. A recent study has shown that downregulation of Napsin A promotes TGF-ß induced cell proliferation in lung adenocarcinoma cells [10].

In diagnostic pathology, Napsin A immunohistochemistry (IHC) is primarily utilized for typing of non-small cell carcinoma, since Napsin A is expressed in a high percentage of adenocarcinomas of the lung (>80%) but only rarely in squamous carcinomas of the lung [3]. When Napsin A was first described around 25 years ago, it was thought to be a lung-specific marker [1], but soon after it was shown that Napsin A is also expressed in kidney tumors [6], compatible with its physiologic expression in proximal renal tubules. Since then, several studies have shown that Napsin A can also be expressed in other tumor types [7, 11, 12]. For example, immunohistochemical Napsin A positivity was found in 0%–52% of clear cell renal cell carcinomas [6, 13–22], 72%–97% of papillary renal cell carcinomas [6, 13, 14, 16–18, 20–22], 0%–48% of thyroid tumors [10, 11, 16, 18, 19, 23, 24], 69%–100% of clear cell carcinomas of the ovary [7, 19, 25–32] and 0%–9% of cholangiocarcinomas [12, 19, 33, 34]. Partially conflicting results with respect to positivity rates between these studies may be due to the use of different antibodies, the use of different immunostaining protocols as well as different criteria to determine positivity in these studies. For many other tumor types, Napsin A expression has never been analyzed.

Since the lung is a frequent site for metastasis of various tumors it is of utmost importance to understand the relative frequency of Napsin A expression in other tumor types and normal tissues. We therefore analyzed Napsin A expression by immunohistochemistry in a tissue microarray format of which 11,957 tumor tissue samples from 115 different tumor types and subtypes as well as 76 non-neoplastic tissue types were evaluable.

## Materials and Methods

### Tissue Microarrays (TMAs)

In order to study Napsin A expression in normal and neoplastic human tissues, preexisting TMAs containing 14,692 primary tumors from 115 tumor types and subtypes were used. Only one core (0.6 mm in diameter) was taken from each tumor. This approach is supported by a large number of TMA studies [35]. The normal tissue microarray contains eight samples respectively from 76 different normal tissues, resulting in a total of 608 spots. All samples were derived from the archives of the Institute of Pathology, University Hospital of Hamburg, Germany, the Institute of Pathology, Clinical Center Osnabrueck, Germany, and the Department of Pathology, Academic Hospital Fuerth, Germany. Tissues were fixed in 4% buffered formalin and then embedded in paraffin. TMA tissue spot diameter is 0.6 mm.

The use of archived remnants of diagnostic tissues for manufacturing of TMAs and their analysis for research purposes as well as patient data analysis has been approved by local laws (HmbKHG, §12) and by the local ethics committee (Ethics commission Hamburg, WF-049/09). All work has been carried out in compliance with the Helsinki Declaration.

### Immunohistochemistry

Freshly cut TMA sections were immunostained on one day and in one experiment. Slides were deparaffinized with xylol, rehydrated through a graded alcohol series and exposed to heat-induced antigen retrieval for 5 min in an autoclave at 121°C in pH nine DakoTarget Retrieval Solution™ (Agilent, CA, United States; #S2367). Endogenous peroxidase activity was blocked with Dako peroxidase Blocking Solution™ (Agilent, CA, United States; #52023) for 10 min. Primary antibody specific against Napsin A protein (mouse monoclonal, MS Validated Antibodies, MSVA-112; Hamburg, Germany) was applied at 37°C for 60 min at a dilution of 1:400. Bound antibody was then visualized using the EnVision Kit™ (Agilent, CA, United States; #K5007) according to the manufacturer’s directions. The sections were counterstained with Hemalaun. This specific antibody was selected because of a favorable signal to noise ratio and its staining pattern in normal tissues coincided with data described in “The Protein Atlas”.

For tumor tissues, the percentage of positive neoplastic cells was estimated, and the staining intensity was semiquantitatively recorded (0, 1+, 2+, 3+). For statistical analyses, the staining results were categorized into four groups. Tumors without any staining were considered as negative. Tumors with 1 + staining intensity in ≤70% of cells and 2 + intensity in ≤30% of cells were considered weakly positive. Tumors with 1 + staining intensity in >70% of cells, 2 + intensity in 31–70%, or 3 + intensity in ≤30% were considered moderately positive. Tumors with 2 + intensity in >70% or 3 + intensity in >30% of cells were considered strongly positive. The analysis was performed by one pathologist (SM).

### Prognostic Evaluation of Napsin A in a Subset of Renal Cell Carcinomas

The tissue specimens were available from patients with renal cell tumors, undergoing surgery between 1994 and 2015 at the Department of Urology, University Medical Center Hamburg-Eppendorf. Detailed histopathological data on ISUP, Fuhrman, and Thoenes grade, UICC, tumor stage (pT), and lymph node metastasis (pN) were available from 575 clear cell and 152 papillary renal cell carcinomas. Clinical follow up data were available from 531 clear cell and 136 papillary renal cell carcinomas with a median follow-up of 40/40months (range 1–250/2–247 months). To thoroughly analyze the potential prognostic value of Napsin A, the subset was separately analyzed at two different antibody dilutions (1:400 and 1:135). This subset of renal cell carcinomas with clinicopathological information has been used in several previously published studies (for example [36–42]).

### Statistics

Statistical calculations were performed with JMP 14 software (SAS Institute Inc., NC, United States). Contingency tables and the chi^2^-test were performed to search for associations between Napsin A and tumor phenotype. Survival curves were calculated according to Kaplan-Meier. Log-Rank test and univariable cox proportional hazard regression was applied to detect significant differences between different Napsin A immunostaining groups. Multivariable cox proportional hazard analysis was performed to test the statistical independence and significance between clinico-patholigcal variables and Napsin A immunostaining in relation to recurrence free survival and cancer specific survival.

## Results

### Technical Issues

A total of 11,957 (81.4%) of 14,692 tumor samples and 500 (82.2%) of 608 normal samples were interpretable for Napsin A immunostaining in our TMA analysis. Non-interpretable samples (2,843; 18.6%) either lacked unequivocal tumor cells or were lost from the TMA during the technical procedures.

### Napsin A in Normal Tissue

**FIGURE 1:**
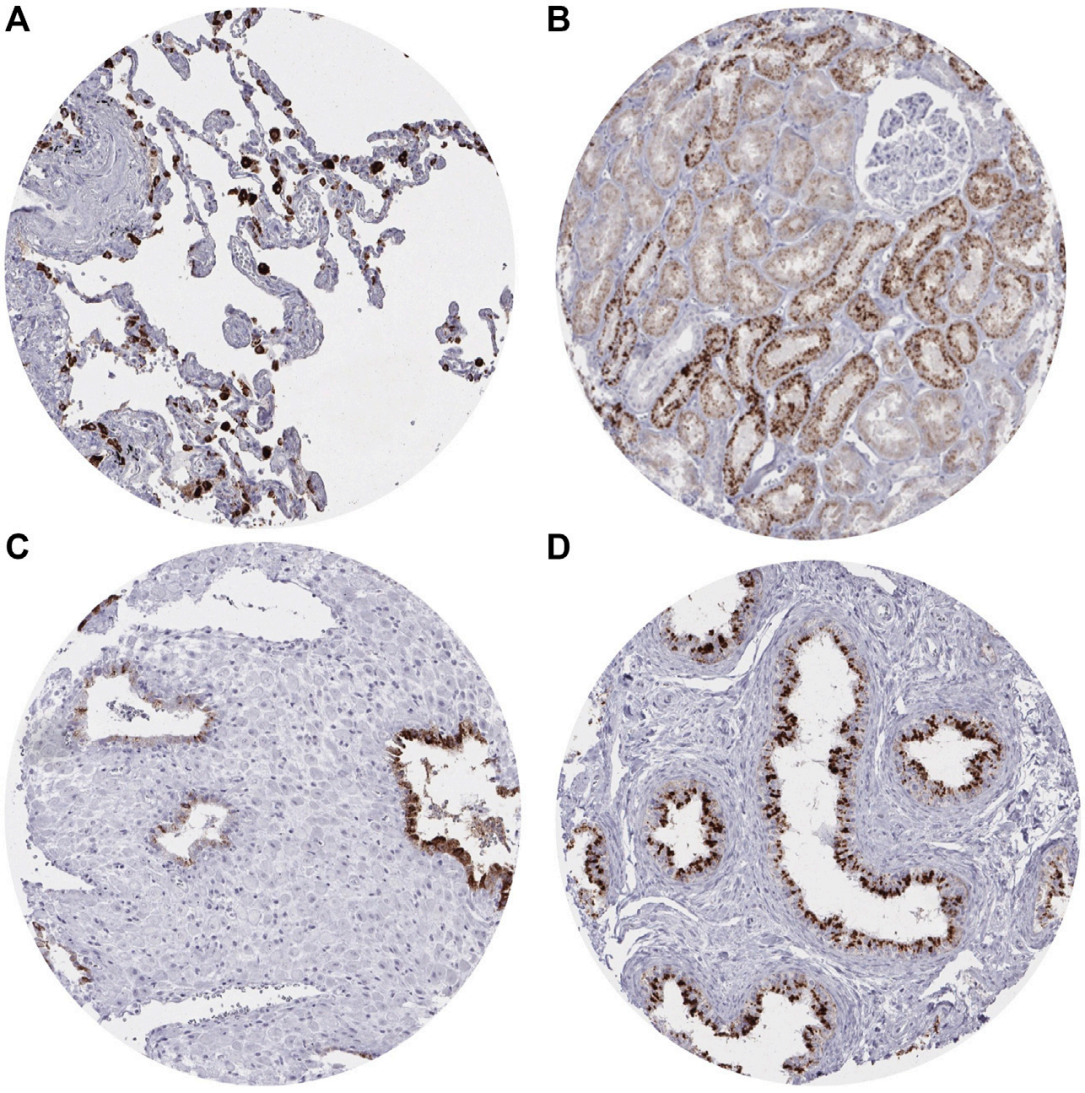
Representative images of Napsin A immunostaining in non-neoplastic tissue.**(A)** Positive staining in pneumocytes in the lung. **(B)** Positive staining in the renal cortex of the kidney (proximal tubules > distal tubules). **(C)** Positive staining in endometrial glands in decidualized stroma. **(D)** Positive staining in the tubules of the epididymis.

A moderate to strong (2+/3+) cytoplasmic Napsin A staining was found in pneumocytes and alveolar macrophages of the lung (Figure 1A), and the renal medulla and cortex of the kidney (proximal > distal) (Figure 1B). In addition, strong cytoplasmic Napsin A staining was found occasionally in endometrial glands in decidualized stroma (Figure 1C), and weak to moderate (1+/2+) staining was found in the tubules of the epididymis (Figure 1D). In contrast to previous studies [5] we did not find any Napsin A staining in seven evaluable spots with normal pancreatic tissue. Napsin A immunostaining was also absent in endothelium and media of the aorta, the heart, striated muscle, tongue muscle, myometrium of the uterus, muscular wall of the appendix, esophagus, stomach, ileum, colon descendens, kidney pelvis, and urinary bladder, corpus spongiosum of the penis, corpus luteum, and follicular cyst of the ovary, ovarian stroma, fallopian tube, fat, skin (including hair follicles and sebaceous glands), oral mucosa of the lip, oral cavity, surface epithelium of the tonsil, transitional mucosa and skin of the anal canal, ectocervix, squamous epithelium of the esophagus, urothelium of the kidney pelvis and urinary bladder, amnion and chorion of the mature placenta, lymph nodes, spleen, thymus, tonsil, mucosa of the stomach (antrum and corpus), duodenum, ileum, appendix, colon descendens, rectum, and gall bladder, liver, parotid, submandibular, and sublingual gland, bone marrow, Brunner gland of the duodenum, prostate, seminal vesicle, testis, mucosa and glands of the bronchus, sinus paranasales, breast, endocervix, proliferative and secreting endometrium, adrenal gland, parathyroid, thyroid gland, stratum moleculare and neuronorum of the cerebellum, white and gray cerebrum, and posterior and anterior lobe of the pituitary.

### Napsin A in Tumor Cells

**FIGURE 2:**
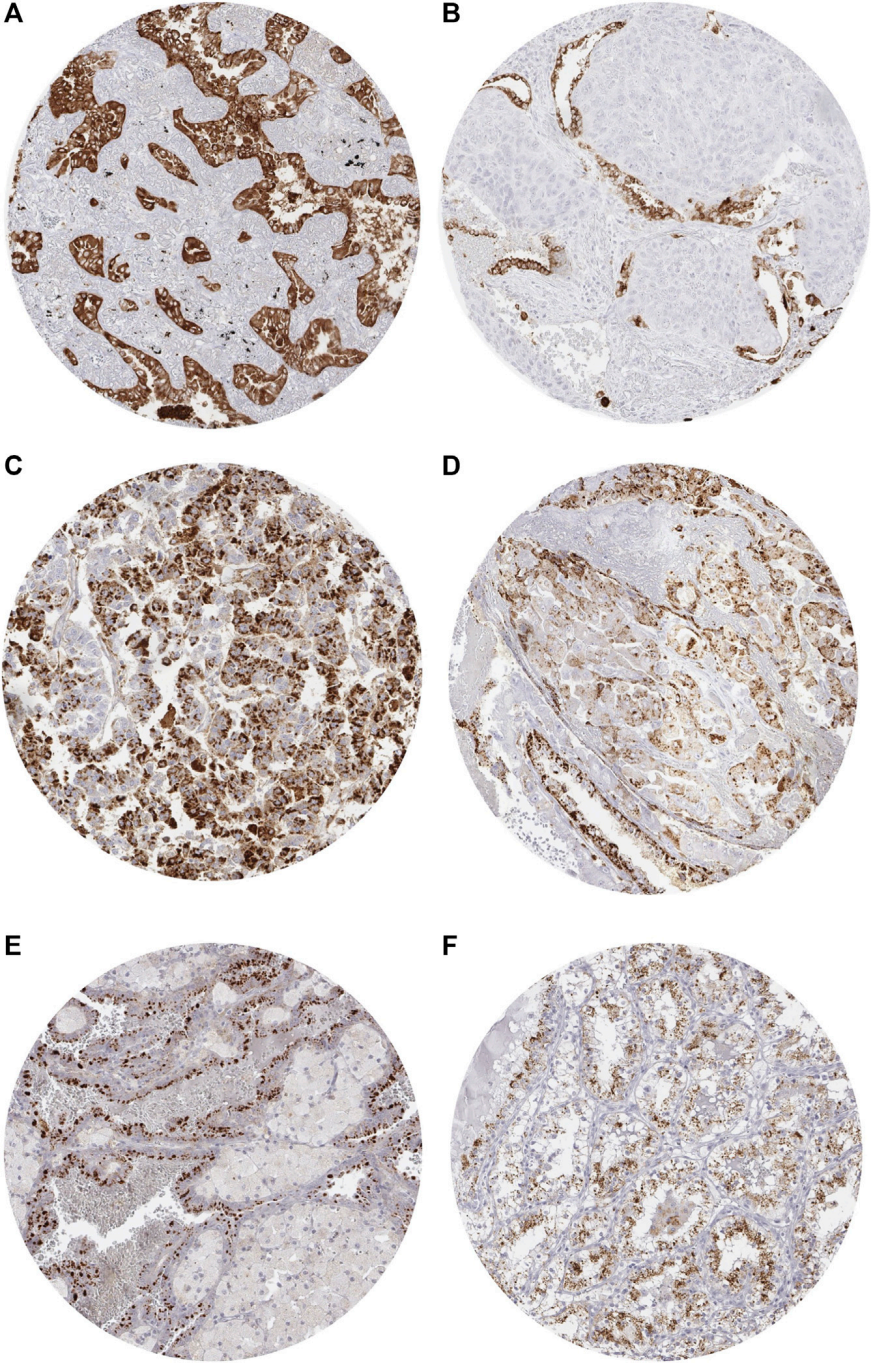
Representative images of Napsin A immunostaining in tumors. **(A)** Positive staining in adenocarcinoma of the lung. **(B)** Absent staining in a squamous carcinoma of the lung with scattered positive pneumocytes. **(C)** Positive staining in clear cell carcinoma of the ovary. **(D)** Positive staining in endometrial clear cell carcinoma. **(E)** Positive staining in papillary renal cell carcinoma. **(F)** Positive staining in clear cell (tubulo) papillary renal cell carcinoma.

**FIGURE 3:**
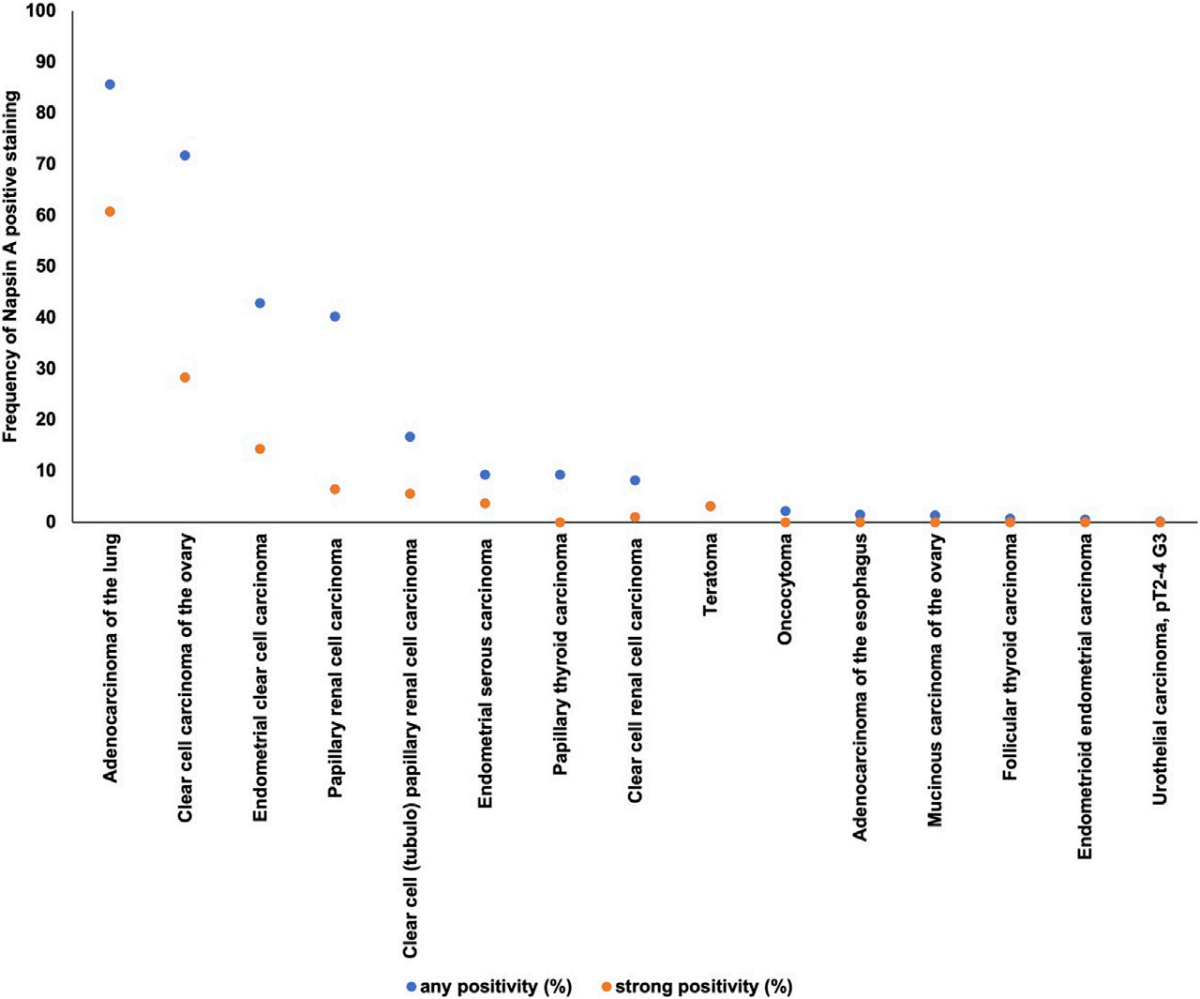
Ranking order of Napsin A immunostaining in human tumors. Both the frequency of positive cases (blue dots) and the frequency of strongly positive cases (orange dots). 98 additional tumor entities without any Napsin A positive cases are not shown due to space restrictions.

Positive Napsin A immunostaining was detectable in 396 (3.3%) of the 11,957 analyzable tumors, including 156 (1.3%) with weak, 82 (0.7%) with moderate, and 158 (1.3%) with strong immunostaining. Representative images of Napsin A positive tumors are shown in Figure 2 and Supplementary Figure S1. Overall, 16 (13.9%) of 115 tumor categories showed a detectable Napsin A expression with 8 (7.0%) tumor categories showing at least in a small proportion of cases strong positivity (Supplementary Table S1). The highest rate of positive staining was found in adenocarcinoma of the lung (85.6%, Figure 2A), clear cell carcinoma of the ovary (71.7%, Figure 2C), endometrial clear cell carcinoma (42.8%, Figure 2D), and papillary renal cell carcinoma (40.2%, Figure 2E). Important tumor types with low or absent Napsin A immunostaining included various squamous cell carcinomas (e.g. lung, larynx, esophagus), different subtypes of breast carcinomas, adenocarcinomas of the prostate, non-invasive papillary urothelial carcinomas, various soft tissue tumors, and bone tumors. A graphical representation of a ranking order of Napsin A positive and strongly positive cancers is given in Figure 3.

### Prognostic Value of Napsin A in Renal Cell Carcinomas

**FIGURE 4:**
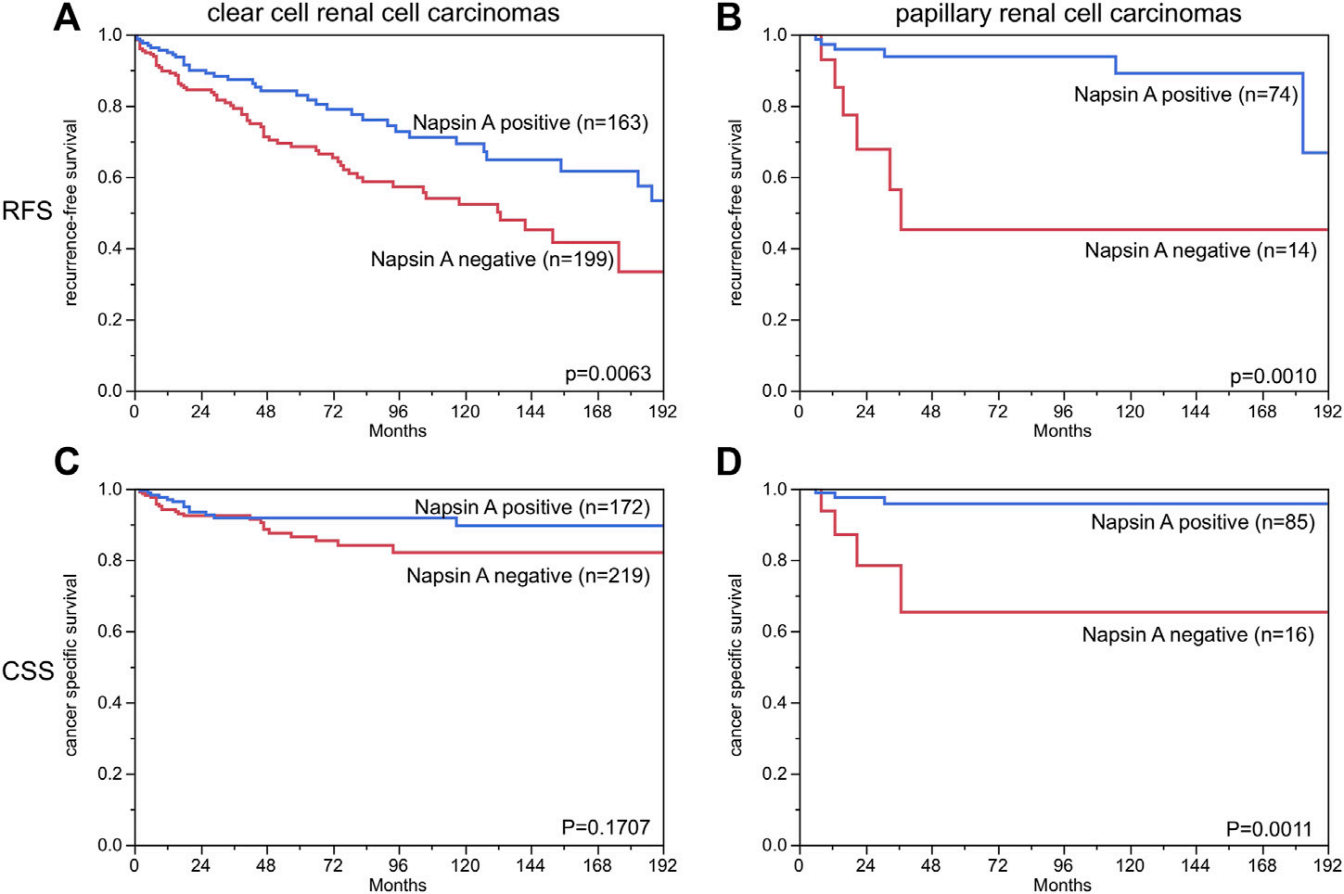
Napsin A immunostaining and recurrence-free survival and cancer specific survival in patients with papillary and clear cell renal cell carcinoma. Weak, moderate and strong staining are combined as “positive”. (RFS = recurrence free survival, CSS = cancer specific survival). *The numbers do not add to the total number of tumors with clinical follow-up data, since only cases with evaluable Napsin A staining are included.

Clear cell and papillary renal cell carcinoma were analyzed at different antibody dilutions. As expected at an antibody dilution of 1:135 the number of positive tumors was higher than at 1:400. In the subgroup of clear cell carcinomas there were 8.4% cases at a dilution of 1:400 compared to 44.0% at a dilution of 1:135. Correspondingly, there were 37.3% positive papillary renal cell carcinomas at a dilution of 1:400 compared to 80.9% at a dilution of 1:135. Representative images of Napsin A staining at different dilutions are given in Supplementary Figure S2. There was no correlation with any clinicopathological features such as tumor stage, tumor grade, lymph node status or with survival or recurrence at a dilution of 1:400, but at a dilution of 1:135 there was a positive correlation with low tumor grade (*p* = 0.0055) and late recurrence (*p* = 0.0063) in the subgroup of clear cell carcinomas and a correlation with low tumor stage (*p* = 0.027), late recurrence (*p* = 0.001) and long tumor-specific survival (*p* = 0.011) in the subgroup of papillary renal cell carcinomas (Table 1; Figure 4). Multivariable analyses showed no independent prognostic relevance from the established clinico-pathological parameters in relation to recurrence free survival and cancer specific survival (Table 2).

**TABLE 1:** Napsin A immunostaining (dilution 1:135) and tumor phenotype of clear cell and papillary renal cell carcinoma.

	Napsin a immunostaining in clear cell renal cell carcinomas						Napsin a immunostaining in papillary renal cell carcinomas					
	n	Negative	Weak	Moderate	Strong	*p* value	n	Negative	Weak	Moderate	Strong	*p* value
All cancers	575	56.0	27.5	8.2	8.3		152	19.1	14.5	12.5	53.9	
ISUP												
1	192	51.0	29.2	9.4	10.4	0.0134	32	15.6	15.6	12.5	56.3	0.5198
2	178	55.1	29.2	6.7	9.0		70	14.3	14.3	12.9	58.6	
3	163	55.8	28.2	9.8	6.1		48	29.2	12.5	10.4	47.9	
4	34	85.3	8.8	0.0	5.9		1	0.0	100.0	0.0	0.0	
Fuhrmann												
1	27	40.7	33.3	14.8	11.1	0.0241	1	0.0	0.0	0.0	100.0	0.2896
2	339	54.0	28.6	8.0	9.4		102	14.7	14.7	12.8	57.8	
3	167	56.3	27.5	9.6	6.6		45	26.7	13.3	11.1	48.9	
4	41	80.5	14.6	0.0	4.9		3	66.7	33.3	0.0	0.0	
Thoenes												
1	205	51.2	28.8	8.3	11.7	0.0055	40	12.5	12.5	15.0	60.0	0.7994
2	316	55.1	28.8	9.2	7.0		102	20.6	15.7	10.8	52.9	
3	53	79.2	15.1	1.9	3.8		9	33.3	11.1	11.1	44.4	
UICC												
1	251	57.0	27.5	7.6	8.0	0.8788	85	14.1	15.3	11.8	58.8	0.0540
2	30	50.0	30.0	6.7	13.3		9	33.3	0.0	22.2	44.4	
3	76	64.5	23.7	6.6	5.3		3	0.0	66.7	0.0	33.3	
4	62	64.5	21.0	8.1	6.5		11	45.5	9.1	0.0	45.5	
Tumor stage (pT)												
1	332	55.1	28.0	7.5	9.3	0.8502	109	12.8	15.6	12.8	58.7	0.0270
2	59	55.9	27.1	6.8	10.2		27	22.2	7.4	18.5	51.9	
3–4	180	57.8	26.7	9.4	6.1		11	54.6	18.2	0.0	27.3	
Lymphnode metastasis (pN)^a^												
0	103	57.3	29.1	4.9	8.7	0.2725	16	25.0	25.0	6.3	43.8	0.2322
≥1	13	61.5	23.1	15.4	0.0		7	42.9	0.0	0.0	57.1	

^a^ Numbers do not always add up to the total number in the different categories because of cases with missing data.

**TABLE 2:** Univariable and multivariable Cox regression analyses.

	Univariable cox regression analyses				Multivariable cox regression analyses			
Parameter	Clear cell renal cell carcinoma		Papillary renal cell carcinoma		Clear cell renal cell carcinoma		Papillary renal cell carcinoma	
	Ep: RFS	Ep: CSS	Ep: RFS	Ep: CSS	Ep: RFS	Ep: CSS	Ep: RFS	Ep: CSS
	*p* value	*p* value	*p* value	*p* value	*p* value	*p* value	*p* value	*p* value
ISUP	<0.0001	<0.0001	0.0015	—	0.2421	0.2241	0.8549	0.8419
Fuhrmann	<0.0001	<0.0001	0.0032	0.3023	0.4526	0.5251	0.8993	0.8670
Thoenes	<0.0001	<0.0001	0.0012	0.0377	0.2295	0.0852	0.2735	0.2806
UICC	<0.0001	<0.0001	<0.0001	<0.0001	0.0550	0.2512	0.0413	1.0000
Tumor stage (pT)	<0.0001	<0.0001	<0.0001	0.0001	0.3931	0.1097	0.0539	0.1933
Lymph node metastasis (pN)	<0.0001	0.0158	0.0046	0.0090	0.3122	0.8636	0.0559	1.0000
Napsin A	0.0061	0.1670	0.0061	0.0083	0.4835	0.3929	0.4313	0.3871

Abbreviations: CSS, Cancer specific survival; EP, endpoint; RFS, Recurrence-free survivial.

## Discussion

Our extensive analysis of 11,957 evaluable tumors from 115 different tumor entities identified 16 tumor types with at least minimal Napsin A expression in at least one case. Tumor entities with highest rates of Napsin A positivity included adenocarcinoma of the lung (85.6%), papillary renal cell carcinoma (40.2%), clear cell adenocarcinoma of the endometrium (42.8%) and the ovary (71.7%) and clear cell (tubulo) papillary renal cell carcinoma (16.7%). Tumor entities found to be potentially Napsin A positive also included three types of neoplasia for which Napsin A expression has not yet been reported, such as follicular thyroid carcinoma (n = 2; 0.7%), urothelial carcinoma of the bladder (n = 2; 0.1%) and teratoma (n = 1; 3.1%).

**FIGURE 5:**
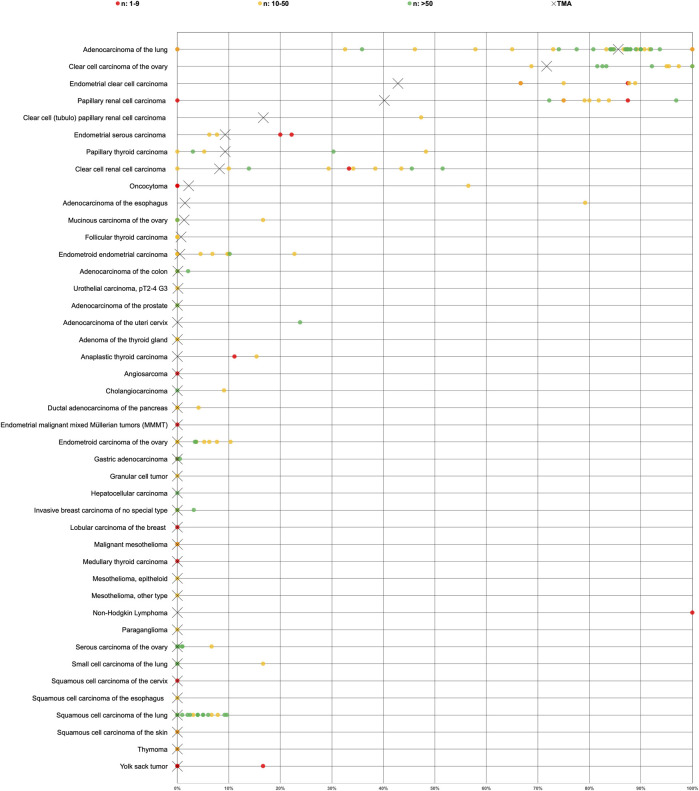
Graphical representation of Napsin A data from this study (marked with a cross) in comparison with the previous literature (marked with a dot). In order to simplify the figure the percentage of weak, moderate and strong staining was merged. Yellow crosses are used for tumor entities with 10–50 evaluable cases and green crosses are used for tumor entities with >50 evaluable cases. Red dots are used for studies from previous studies involving 1–9 cases, yellow dots for studies involving 10–50 cases and green dots for studies involving >50 cases. All studies are quoted in the list of references.

More than 70 studies have previously analyzed Napsin A expression in tumors by IHC. The studies showed a wide range of Napsin A positivity for each tumor entity, for example, published Napsin A positivity rates ranged from 0% to 100% in adenocarcinoma of the lung [11, 13, 16, 18, 20, 22, 24, 43–66], 0%–48% in papillary thyroid carcinoma [11, 16, 18, 19, 24], 0%–52% in clear cell renal cell carcinoma [6, 13, 15–19, 22, 24], 0%–17% in small cell carcinoma of the lung [13, 22, 50, 61, 63], 0–10% in squamous cell carcinoma of the lung [16, 18, 22, 24, 46–48, 50, 52, 57–59, 61–65, 67–69], 69–100% in clear cell adenocarcinoma of the ovary [7, 19, 25, 27–32, 34], 72%–97% in papillary renal cell carcinoma [6, 16–18, 20–22] and 67%–89% in clear cell adenocarcinoma of the endometrium [7, 30, 70, 71]. The analysis of a large number of different tumor entities under highly standardized conditions enabled us to clarify the relative importance of Napsin A expression across tumor entities and to generate a ranking list according to the expected rate of Napsin A positivity. In Figure 5, the data of previous studies are summarized and compared with data from our study.

In tumor pathology, Napsin A IHC is mainly used for subtyping of lung tumors and for differentiating ovarian high grade carcinomas. Major therapeutic advances have been made in recent years in the lung cancer field with different therapeutic strategies for different tumor types, making it inevitable for the pathologist to make an exact diagnosis on a small biopsy. A strong role of Napsin A in the difficult distinction of adenocarcinoma and squamous cell carcinoma in the lung is strongly supported by our Napsin positivity of 85.6% in 198 adenocarcinomas, while none of our 79 squamous cell carcinomas were found positive. That none of 296 analyzed squamous cell carcinomas from other organs of origin were Napsin A positive further emphasizes that this protein is virtually absent in cells with squamous differentiation. It is of note that other investigators have reported Napsin A positivity in 0–10% of pulmonary squamous cell carcinomas in studies analyzing 14–569 tumors [16, 18, 22, 24, 46–48, 50, 52, 57–59, 61–65, 67–69]. One possible reason for a perceived Napsin A positivity in squamous cell carcinomas that we and others encountered is entrapped normal lung tissue with Napsin A positive hyperplastic pneumocytes or Napsin A positive intra-alveolar macrophages between cancer cells [3]. Our finding of 71.7% Napsin A positive clear cell ovarian carcinomas while none of 521 serous high grade carcinomas were Napsin A positive further corroborates the previously suggested diagnostic utility of Napsin A IHC for the distinction of these tumors [27]. A limitation of our study is the absence of large cell carcinomas of the lung which have not been included on the tissue microarray.

To differentiate adenocarcinoma from the lung from pulmonary metastases of extrapulmonary origin and to support a pulmonary origin of metastases from unknown primary tumors is another major application of Napsin A IHC. The virtual limitation of Napsin A expression to few cancer types makes Napsin A highly useful marker for assessing the site of origin of cancers. It is a potential pitfall, however, that several cancer types, that often metastasize to the lung belong to the exclusive group of potentially Napsin A positive cancers, such as renal cell carcinoma, urothelial cancer, colorectal carcinoma and clear cell carcinomas of the ovary and the endometrium. Approximately one third of patients with renal cell carcinoma present with metastatic disease, in most cases metastasis to the lung [72]. Therefore, a biopsy of a mass in the lung could, on the basis of Napsin A positivity, be misdiagnosed as a primary adenocarcinoma of the lung. Several case reports have indeed reported such unfortunate cases [73–75].

All our data are based on the analysis of TMA spots measuring 0.6 mm in diameter. TMAs are thus highly suited to model the diagnostic situation in small biopsies such as bronchial biopsies where the tumor cell content is comparably small. That we observed a similarly high rate of Napsin A positivity (85%) as found by most other studies using larger tissue samples [16, 24, 49, 50, 52, 58, 59, 61, 63] suggests a low rate of Napsin A expression heterogeneity in adenocarcinomas. It is well possible, that the analysis of larger specimen would result in somewhat higher positivity rate in squamous cell carcinomas than the 0% in our study. This might either be due to a higher risk of entrapped normal Napsin A positive macrophages or pneumocytes mimicking Napsin A positivity or to true focal expression in tumor cells. In this context, it is of note, that the only study comparing immunostaining data obtained on TMAs vs. findings in corresponding large sections with patient prognosis as the study endpoint found a superiority of TMA data. Although the large section analysis of more than 500 breast cancers had identified almost twice as many p53 positive cases (40%) than each of four different TMAs containing one spot each per tumor (20% each), all four TMAs–but not the large section data - identified a strong prognostic impact of p53 positivity [76]. Torhorst et al [76] concluded from their data that either staining artifacts or clinically irrelevant focal p53 alterations were responsible for their unexpected results.

In this study, the prognostic role of Napsin A expression was evaluated in renal cell carcinomas because this tumor cohort included significant numbers of Napsin A positive and negative cases. The most noticeable result of this analysis is the dependency of the study outcome on the selected antibody dilution. While significant differences in outcome were not visible at a dilution of 1:400, the number of positive cases increased at a dilution of 1:135 and significant differences became visible. The reason for decreased prognosis in renal cell carcinomas with a Napsin A expression loss is unclear. Previous studies analyzing drug resistance in lung cancer cells suggested that Napsin A expression may inhibit epithelial-mesenchymal transition (EMT) [77, 78], which apart from being a mechanism for resistance to chemotherapy could play a role in tumor progression. However, many physiological functions of renal tubular cells are no longer required in renal tumors. It is thus also possible, that the loss of physiological Napsin A expression is a bystander phenomenon occurring as a result of a general dedifferentiation of tumor cells during cancer progression and which does not exert cancer-relevant effects on cell function. There is only a small number of previous studies analyzing the prognostic value of Napsin A expression in cancer. Multiple studies had suggested that Napsin A expression may be a predictor for prolonged overall survival in adenocarcinoma of the lung [55, 60, 79, 80]. In another study Fadare et al [71], did not find associations of Napsin A expression and survival or clinicopathological features in clear cell carcinomas of the endometrium. Considering, that the prognostic impact of Napsin A expression was rather weak and only visible at a high antibody concentration that already resulted in considerable background staining, we do not anticipate a major role of Napsin A analysis for kidney cancer prognosis assessment.

## Conclusion

These results provide a comprehensive overview on Napsin A expression in human cancers and a systematic comparison with previous studies. The data also show that a reduced or lost Napsin A expression is linked to tumor dedifferentiation in renal cell carcinomas.

## Data Availability

The raw data supporting the conclusions of this article will be made available by the authors, without undue reservation.

## Abbreviations

CSS, cancer specific survival; EMT, epithelial-mesenchymal transition; IHC, Immunohistochemistry; ISUP, International Society of Uropathology; Napsin A, Novel aspartic proteinase of the pepsin family A; RFS, recurrence free survival; TMA, tissue microarray; TTF1, thyroid transcription factor 1; UICC, Union internationale contre le cancer.
