# Supplementary figures and images for: Napsin A Expression in Human Tumors and Normal Tissues
Source: Pathol Oncol Res. 2021 Apr 20;27:613099. doi: 10.3389/pore.2021.613099 (PMC8262149; doi:10.3389/pore.2021.613099)

a)

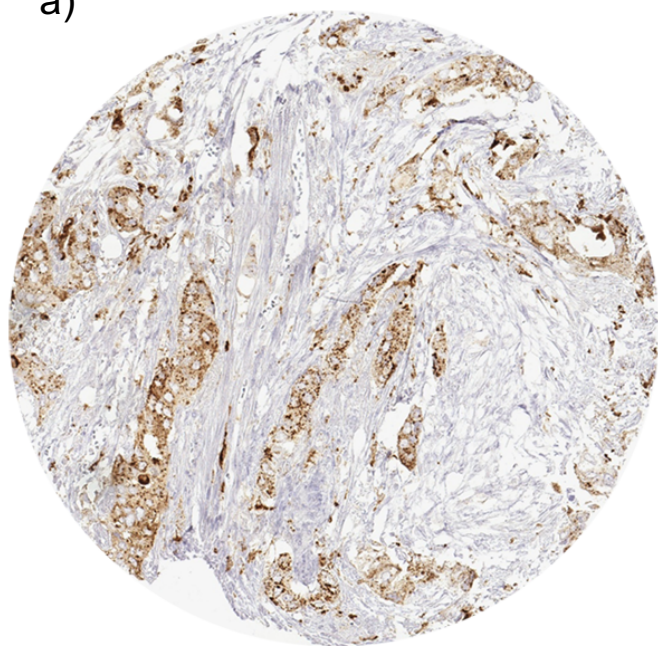

b)

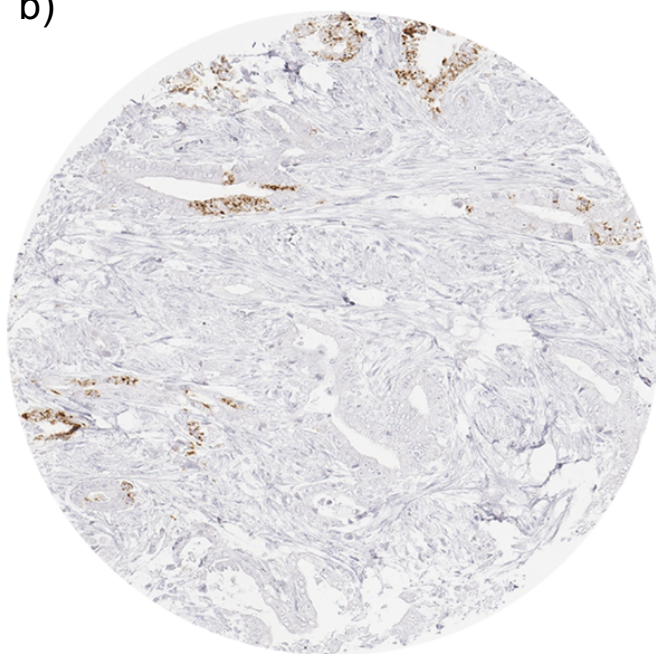

c)

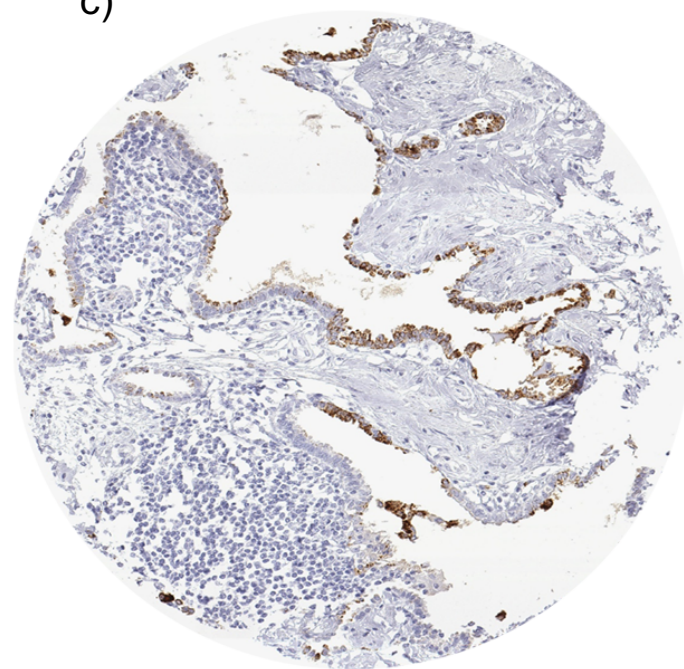

Supplement: Supplementary file 1 [file DataSheet1.PDF]

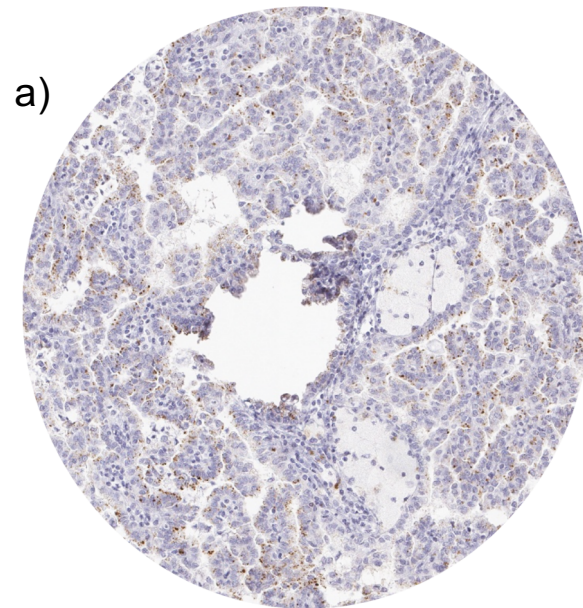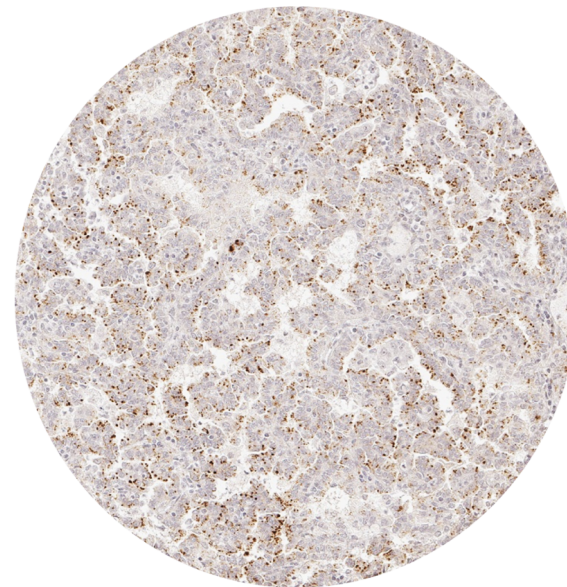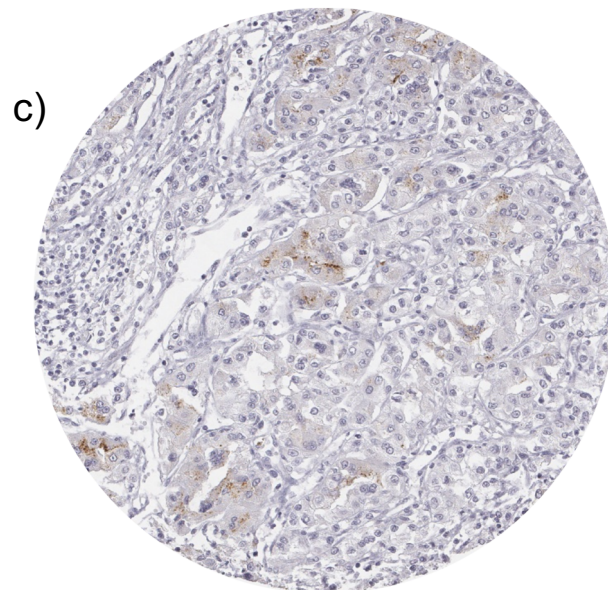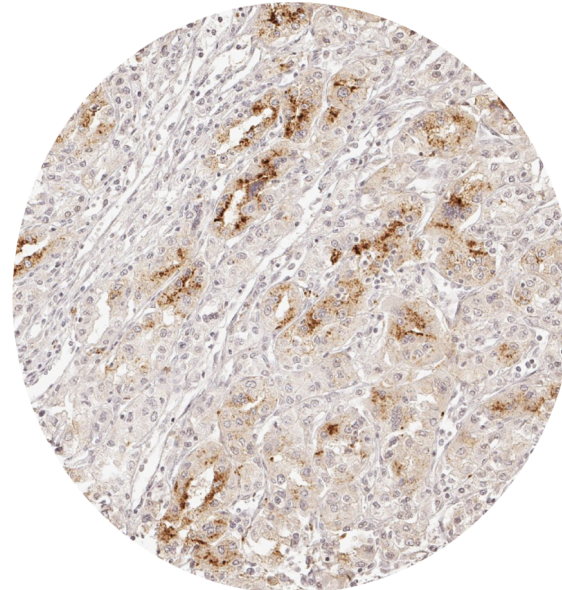

Supplement: Supplementary file 2 [file DataSheet2.PDF]
